# Impact of Zostavax Vaccination on T-Cell Accumulation and Cutaneous Gene Expression in the Skin of Older Humans After Varicella Zoster Virus Antigen–Specific Challenge

**DOI:** 10.1093/infdis/jiy420

**Published:** 2018-09-22

**Authors:** Neil P Patel, Milica Vukmanovic-Stejic, Mayte Suarez- Farinas, Emma S Chambers, Daisy Sandhu, Judilyn Fuentes-Duculan, Neil A Mabbott, Malcolm H A Rustin, James Krueger, Arne N Akbar

**Affiliations:** 1 Division of Infection and Immunity, University College London; 2 Department of Dermatology, Royal Free Hospital, London; 3 Roslin Institute, University of Edinburgh, Midlothian, United Kingdom; 4 Royal (Dick) School of Veterinary Studies, University of Edinburgh, Midlothian, United Kingdom; 5 Laboratory for Investigative Dermatology, Rockefeller University, New York, New York

**Keywords:** Zostavax, cutaneous immunity, resident memory T cells, aging

## Abstract

**Background:**

The live attenuated vaccine Zostavax was developed to prevent varicella zoster virus (VZV) reactivation that causes herpes zoster (shingles) in older humans. However, the impact of vaccination on the cutaneous response to VZV is not known.

**Methods:**

We investigated the response to intradermal VZV antigen challenge before and after Zostavax vaccination in participants >70 years of age by immunohistological and transcriptomic analyses of skin biopsy specimens collected from the challenge site.

**Results:**

Vaccination increased the proportion of VZV-specific CD4^+^ T cells in the blood and promoted the accumulation of both CD4^+^ and CD8^+^ T cells in the skin after VZV antigen challenge. However, Zostavax did not alter the proportion of resident memory T cells (CD4^+^ and CD8^+^) or CD4^+^Foxp3^+^ regulatory T cells in unchallenged skin. After vaccination, there was increased cutaneous T-cell proliferation at the challenge site and also increased recruitment of T cells from the blood, as indicated by an elevated T-cell migratory gene signature. CD8^+^ T-cell–associated functional genes were also highly induced in the skin after vaccination.

**Conclusion:**

Zostavax vaccination does not alter the abundance of cutaneous resident memory T cells but instead increases the recruitment of VZV-specific T cells from the blood and enhances T-cell activation, particularly cells of the CD8^+^ subset, in the skin after VZV antigen challenge.

Older individuals show reduced immune function, leading to an increased incidence of infection and malignancy [1, 2]. The increased susceptibility to infection in this population is complicated by less robust immunological responses to vaccination [3]. As the number of people in the world aged 60 years and over will almost triple to 2 billion by 2050, the consequences for infection-related morbidity and mortality and the resource implications for healthcare delivery are profound.

Varicella zoster virus (VZV) causes the primary infection varicella (or chickenpox). Following resolution of the primary infection, the virus maintains lifelong latency in dorsal root ganglia. Reactivation of VZV, known as herpes zoster (HZ) or shingles, is associated with age- or disease-induced waning of VZV-specific cellular immunity [4–6]. While it is clear that T-cell–mediated immunity plays a crucial role in immune protection against reactivation of VZV, as indicated by an increased incidence of HZ in immunosuppressed individuals [6, 7], the mechanisms of this protection have not been elucidated. Cutaneous T-cell immune responses to intradermal VZV antigen challenge are considered to reflect the status of an individual’s immune response to this pathogen [8, 9]. A weak skin immune response to VZV antigen challenge is associated with a greater susceptibility to HZ and postherpetic neuralgia [10, 11]. Cutaneous immunity is observed to wane with increased age [12], but the mechanisms responsible for this decline are unknown; it is also unclear whether the deterioration in skin immunity is responsible for the lack of protection against VZV reactivation during aging.

The live attenuated shingles vaccine, with the proprietary name of Zostavax, was developed to prevent HZ in older individuals [13]. The vaccine has been shown to boost the robustness of circulating VZV-specific CD4^+^ T-cell responses and VZV-specific antibody production, but its effects on VZV-specific T cells in the skin are not known [14, 15]. As the skin is the first site to which the virus migrates after reactivation in the dorsal root ganglia [16], we sought to elucidate how Zostavax vaccination alters immune cell populations resident within the skin.

We previously developed an experimental system to investigate antigen-specific immunity in vivo, whereby healthy volunteers are challenged intradermally with a recall antigen to induce an antigen-specific delayed-type hypersensitivity response [17]. Using this experimental system, we identified that older humans (age, >65 years) demonstrate decreased cutaneous immune responses to VZV antigen challenge [12]. In this current study, we investigated the impact of Zostavax vaccination on cutaneous immunity to VZV in older individuals. A cohort of volunteers aged >70 years was recruited, and their responses to VZV antigen challenge were assessed before and after vaccination. We show that, although vaccination increased the proportion of VZV-specific CD4^+^ T cells in the blood, the proportion of these cells within the skin (ie, skin-resident memory T cells [T_RM_]) was not affected. Vaccination also enhanced the skin response to VZV antigen challenge and was associated with increased T-cell accumulation and proliferation at the site of antigen challenge. After vaccination, there was a marked upregulation of genes associated with migration and activation of T cells at the site of antigen challenge. Additionally, there was a pronounced expression of a CD8^+^ T-cell–related gene signature in the skin after VZV antigen challenge in vaccinated individuals. Taken together, these data suggest that the improved VZV-specific cutaneous immunity after Zostavax vaccination is due to the recruitment of VZV-specific T cells from the blood that are then activated and induced to proliferate in the skin.

## MATERIALS AND METHODS

This work was approved by the Ethics Committee of Queen’s Square (London) and by the Institutional Review Board of the University College London Research and Development Office. We recruited 122 healthy older individuals (age, >70 years; median age, 75 years; 77 women and 45 men) and 108 healthy young individuals (age, <40 years; median age, 28 years; 60 women and 48 men) into the study investigating the effects of age on immunity to VZV.

**Figure 1.**
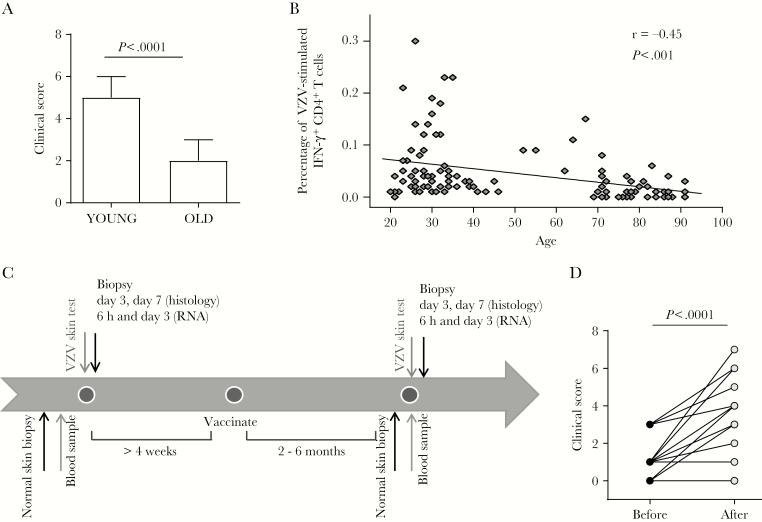
Zostavax vaccination improves cutaneous response to varicella zoster virus (VZV) antigen challenge in older individuals. *A*, Healthy young (n = 108) and older (n = 122) volunteers were injected with 0.02 mL of VZV skin antigen test. A clinical score at day 3 was calculated based on induration, palpability, and redness. *B*, Proportion of VZV-specific CD4^+^ T cells (defined as being positive for interferon γ [IFN-γ^+^] after overnight stimulation with VZV lysate) in peripheral blood of young and older volunteers (n = 104; 55 young, 12 middle-aged, and 37 older adults). *C*, Schematic representation of the study to investigate the effects of Zostavax on VZV antigen challenge in older individuals (n = 30). *D*, Change in day 3 clinical score before and after vaccination. For panels *A* and *D*, data were assessed by Mann-Whitney and Wilcoxon paired tests, respectively. For panel *B*, data were assessed by a Pearson correlation test.

For the vaccination study, we recruited a subset of 30 healthy older individuals aged >70 years (age range, 70–93 years; median age, 75.5 years; 10 men and 20 women). Following the initial VZV skin antigen challenge, Zostavax was administered as per the manufacturer’s instructions to individuals whose clinical score was ≤4. Between 2 and 6 months later, VZV antigen challenge was repeated, as depicted in the schematic representation of the study in Figure 1C. Individuals with a history of neoplasia, immunosuppressive disorders, or inflammatory skin disorders were excluded from this investigation. Furthermore, we excluded individuals with comorbidities that are associated with significant internal organ or immune dysfunction, including heart failure, severe chronic obstructive pulmonary disease, diabetes mellitus, and rheumatoid arthritis. We also excluded individuals receiving immunosuppressive regimens for the treatment of autoimmune or chronic inflammatory diseases (eg, oral glucocorticoids, methotrexate, azathioprine, and cyclosporine). However, we did not exclude older individuals who take aspirin, nonsteroidal antiinflammatory drugs, statins, and antihypertensive agents, owing to the widespread use of these drugs in this age group. All volunteers provided written, informed consent, and study procedures were performed in accordance with the principles of the Declaration of Helsinki.

### Skin Tests

VZV antigen (Biken, The Research Foundation for Microbial Diseases of Osaka University, Japan) was injected intradermally into the sun-protected skin of the medial proximal volar forearm as per the manufacturer’s instructions. Induration, palpability, and the change in erythema from baseline were measured and scored on day 3 as described previously [17]. A clinical score (range, 0–10) based on the summation of these parameters was then calculated [17]. Older individuals with a clinical score >4 were excluded from further participation, as such individuals showed characteristics of cellular infiltration and responsiveness indistinguishable from those observed in young volunteers [8, 18].

### Skin Biopsy Specimens

For immunofluorescence experiments, punch biopsy specimens (5 mm in diameter) from the site of VZV antigen challenge were obtained at various time points (as indicated) after injection. Control skin punch biopsy specimens from normal (unchallenged) forearm skin were also obtained. Biopsy specimens were frozen in optimal cutting temperature compound (Bright Instruments). Six-micrometer sections were cut, left to dry overnight, fixed in ethanol and acetone, and stored at −80°C.

### Immunofluorescence

Sections were stained with optimal dilutions of primary antibodies, followed by an appropriate secondary antibody conjugated to various fluorochromes as described elsewhere [8, 12] (Table 1).

**Table 1.** List of Antibodies Used for Immunofluorescence

Antibody name	Clone	Company
CD4	SK3 or YNB46.1.8	BD Bioscience
CD8	RPA-T8	BD Bioscience
Ki67-FITC	B56	BD Bioscience
CD31-FITC	WM59	BD Bioscience
CD69	FN50	Biolegend
CD103	2G5.1	Thermofisher
Foxp3-biotin	PCH101	eBioscience
E-selectin	ENA1	Abcam

Activated endothelium images were acquired on the AxioScan Z1 slide scanner and analyzed on Zen Blue (Zeiss, Cambridge, United Kingdom). All other images were acquired using appropriate filters of a Leica DMLB microscope with a Leica N PLAN 20×/0.40 objective and a Cool SNAP-Pro cf Monochrome Media Cybernetics camera, controlled by Image-Pro Plus 6.2 software. When counting the numbers of cells in perivascular infiltrates, the 5 largest perivascular infiltrates present in the upper and mid-dermis were selected for analysis, and an average was calculated as described previously [19].

### Peripheral Blood Mononuclear Cell (PBMC) Preparation and Skin Biopsy Specimen Digestion

Heparinized blood samples were collected from volunteers at the time of VZV antigen challenge. PBMCs were prepared by density centrifugation on Ficoll-Paque (Amersham Biosciences, Little Chalfont, UK) and resuspended in complete medium.

Skin biopsy specimens (5 mm in diameter) were collected from unchallenged skin and disaggregated by overnight incubation (37°C; 5% CO_2_) in 0.8 mg/mL collagenase IV (Sigma Aldrich) with 20% fetal calf serum. Single-cell suspensions were obtained by filtering the suspension through 100-, 70-, and 40-µm filters.

### Flow Cytometric Analysis

PBMCs or skin-derived leukocytes were stimulated with 40 μL/mL VZV lysate (Virusys, Taneytown, MD) or 1 μL/mL SEB as a positive control and were incubated for 15 hours at 37°C and 5% CO_2_ in the presence of 5 µg/mL brefeldin A (Sigma-Aldrich, Gillingham, UK). Following stimulation, cells were stained using combinations of antibodies, including CD3 (clone UCHT1), CD4 (RPA-T4), and CD8 (SK1), for 30 minutes at 4°C and washed, fixed, and permeabilized (Fix & Perm Cell Permeabilisation Kit, Invitrogen, Paisley, UK) before staining for interleukin 2 (clone 5344.111), interferon γ (IFN-γ; B27), and tumor necrosis factor α (TNF-α; Mab11; all from BD Biosciences, Oxford, UK). Multiparameter investigation of skin and blood T-cell phenotypes was performed on an LSR II or BD Fortessa instrument, using FACS Diva software (BD Biosciences, Oxford, UK), and data were subsequently analyzed using FlowJo, version X (TreeStar, Ashland, OR), as previously described [12, 19].

### Transcriptional Analysis

Punch biopsy specimens (3 mm in diameter) were collected from the VZV antigen challenge site (6 or 72 hours after injection), immediately frozen in RNAlater, and stored at −20°C until use. Unchallenged forearm skin was collected as a control from a number of volunteers. Frozen tissue specimens were homogenized, and total RNA was extracted from bulk tissue homogenates, using the RNeasy Mini Kit (Qiagen). Target amplification and labeling were performed according to standard protocols, using the Nugen Ovation WB Kit. RNA was hybridized to Affymetrix Human Genome U133 2.0 plus arrays. Affymetrix gene chips were scanned for spatial artifacts, using the Hirshlight package [20]. Gene expression measures were obtained using the GCRMA algorithm [21]. Gene expression was modeled using mixed models in R’s limma framework, which accounts for the within-patient correlation structure, and batch effect was eliminated using linear models. Differences between groups were estimated from this model and its significance assessed using the moderated (paired/unpaired) *t* test. Resulting *P* values were adjusted for multiple hypotheses, using the Benjamini-Hochberg procedure. Gene set variation analysis was used to obtain the per-pathway scores for each patient and sample, using a collection of skin-specific pathways curated by the laboratory of J. K. and extensively used in publications [21–23] and the collection of canonical pathways from the Molecular Signatures database (MSigDB; available at: http://software.broadinstitute.org/gsea/msigdb/). Pathway scores were then analyzed with the same approach as the gene-based analysis.

### Statistics

Statistical analysis was performed using GraphPad Prism, version 6.00 (GraphPad Software, San Diego, CA). Paired or unpaired *t* tests were used when data were normally distributed; otherwise, nonparametric tests were used. The Kruskal-Wallis test was used to compare ≥3 unpaired groups, and a 2-tailed Mann-Whitney test was used when comparing only 2 unpaired groups. The Wilcoxon matched pairs test was used when comparing 2 groups of matched data.

## RESULTS

### Decreased Cutaneous Response to VZV Antigen Challenge in Older Individuals Can Be Boosted by Zostavax Vaccination

We confirmed previous observations that older individuals (n = 122; age, >70 years; age range, 70–92 years; 45 men and 77 women) have decreased cutaneous responsiveness to VZV antigen challenge when compared to younger individuals (n = 108; age, <40 years; age, range, 20–39 years; 48 men and 60 women; Figure 1A) [12, 22]. We also confirmed previous findings of a decreased proportion of VZV-specific CD4^+^ T cells in the circulation during aging (Figure 1B); VZV-specific CD4^+^ T cells were defined as IFN-γ–producing leukocytes, after overnight stimulation with VZV lysate in vitro [8, 23].

To investigate the effects of Zostavax on cell-mediated immunity to VZV, we designed a study to compare cutaneous responses in older individuals before and after vaccination (Figure 1C). VZV antigen challenge was performed before and after vaccination, and the clinical score was calculated at 72 hours, as described previously [17]. Of 30 volunteers who consented to the study, 5 had a starting score of 4, reflecting a moderate response; none of these 5 received a boost in clinical response after vaccination.

Further analysis was performed on the remaining 25 volunteers who had a starting clinical score ≤3. Nineteen of 25 (76%) had an enhanced clinical response after vaccination, whereas the remaining individuals (24%) showed no improvement. As a group, there was a significant increase in clinical score after vaccination, from a median score of 1 before vaccination to a median score of 3 after vaccination (*P* < .0001; Figure 1D). In parallel studies, we showed that the rechallenge of older volunteers with VZV skin test antigen at intervals of 2–3 months did not by itself boost their original clinical score [18].

### Effect of Zostavax Vaccination on Skin-Resident Memory T Cells

**Figure 2.**
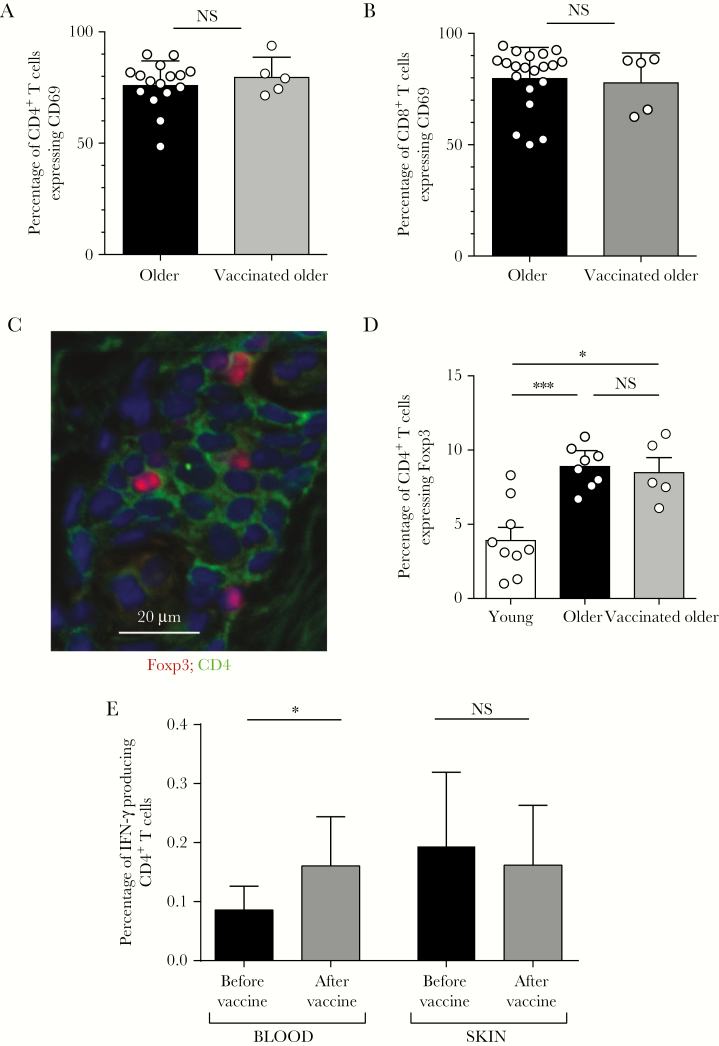
Zostavax vaccination leads to an increase in circulating but not skin-resident varicella zoster virus (VZV)–specific T cells. Punch biopsy specimens (diameter, 5 mm) were taken from unchallenged skin before and after vaccination and immunohistological assessment was performed. *A* and *B*, Frequency of CD4^+^ (*A*) and CD8^+^ (*B*) T cells that expressed CD69. *C* and *D*, Representative immunostaining of CD4 (green) and Foxp3 (red; *C*) and cumulative data showing the frequency of Foxp3^+^ CD4^+^ T cells (*D*) in young and older individuals (before and after vaccination). *E*, Peripheral blood and skin-derived VZV-specific CD4^+^ T cells were enumerated by intracellular cytokine staining following overnight stimulation with VZV lysate (n = 6 for skin and n = 10 for blood). For panels *A* and *B*, data were assessed by the Mann-Whitney test. For panel *D*, data were assessed by 1-way analysis of variance with the Tukey post hoc test. For panel *E*, data were analyzed by the Wilcoxon paired test. Abbreviation: NS, not significant. *P < .05 and **P < .01.

Tissue-resident memory T cells (T_RM_) in the skin and other tissues can be identified by the expression of CD69 [8, 24, 25]. Zostavax vaccination had no effect on the proportions of cutaneous T_RM_ in unchallenged skin, in either CD4^+^ (Figure 2A) or CD8^+^ T-cell subsets (Figure 2B). In addition, T_RM_ can be further subdivided by their relative expression of CD103 [26], but there were also no differences in T-cell CD103 expression before and after Zostavax vaccination (data not shown). As reported previously, older individuals have increased proportions of CD4^+^Foxp3^+^ regulatory T cells (Tregs) in the skin as compared to young individuals [19] (Figure 2C, 2D). However, Zostavax vaccination did not alter the proportions of these cells in unchallenged skin (Figure 2D).

We next investigated the impact of Zostavax vaccination on skin and peripheral blood VZV-specific CD4^+^ T cells. There was a significant increase in the proportion of IFN-γ–producing VZV-specific CD4^+^ T cells in the blood after vaccination (*P* = .027), confirming previous results [15, 27, 28]. In contrast, there was no increase in the proportion of VZV-specific CD4^+^ T cells in unchallenged skin after vaccination (Figure 2E).

### Enhanced Responsiveness to VZV Antigen Challenge After Zostavax Vaccination

**Figure 3.**
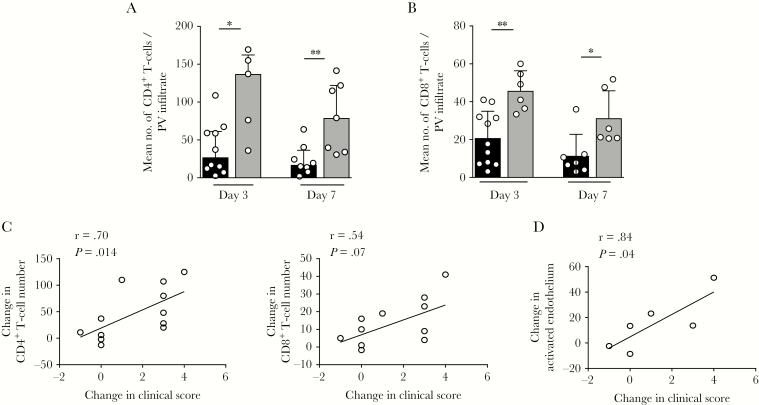
Zostavax vaccination leads to an increase in T-cell accumulation at the site of varicella zoster virus (VZV) antigen challenge. Punch biopsy specimens (diameter, 5 mm) were obtained on days 3 and 7 after VZV antigen challenge, before and after vaccination. *A* and *B*, Cumulative data showing number of CD4^+^ (*A*) and CD8^+^ (*B*) T cells per perivascular (PV) infiltrate before (black) and after (gray) vaccination on days 3 and 7 after VZV antigen challenge. *C*, Correlation between change in CD4^+^ (left) and CD8^+^ (right) T-cell number and change in clinical score after vaccine administration. *D*, Correlation between change in activated endothelium (calculated as the percentage of CD31^+^ loops expressing E-selectin) and change in clinical score after vaccine administration. For panels *A* and *B*, data were assessed by the Mann-Whitney test. For panels *C* and *D*, data were assessed by the Pearson correlation test.

To investigate how Zostavax vaccination altered the kinetics of the VZV-specific immune response in the skin, punch biopsy specimens were collected at the site of VZV antigen challenge at days 3 and 7 after injection, before and after vaccination, and the numbers of CD4^+^ and CD8^+^ T cells in the skin were quantified by immunohistological assessment. The 5 largest perivascular infiltrates in the mid-dermis and upper dermis were chosen and the mean number of leukocytes within them calculated. We previously showed that, on days 3 and 7 following VZV antigen challenge, there were reduced numbers of CD4^+^ T cells present at the challenge site in older as compared to young volunteers [12]. In the current study, we found that, following Zostavax vaccination, older individuals had significantly increased numbers of both CD4^+^ T cells (Figure 3A) and CD8^+^ T cells (Figure 3B) in the skin after VZV antigen challenge. There was a significant correlation between the increase in clinical score after vaccination and the abundance of CD4^+^ T cells in the skin (Figure 3C). Furthermore, there was increased activation of endothelial loops (identified as CD31^+^ E-selectin–positive cells [29]) in the skin at the site of VZV antigen challenge after vaccination (Figure 3D), which was also significantly associated with the increase in clinical score after vaccination.

### Increased T-Cell Proliferation After Zostavax Vaccination in VZV Antigen–Challenged Skin

**Figure 4.**
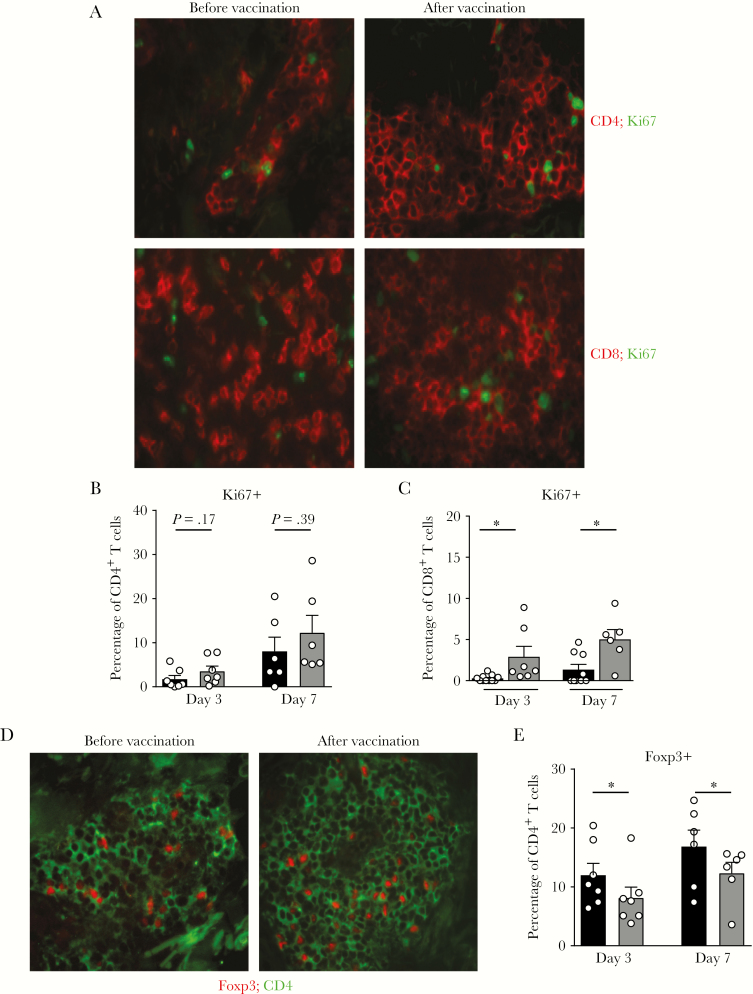
Increased CD8^+^Ki67^+^ T cells at the site of varicella zoster virus (VZV) antigen challenge. Punch biopsy specimens (diameter, 5 mm) were performed on days 3 and 7 after VZV antigen challenge, before and after vaccination. *A*, Representative images showing the frequency of CD4^+^ (top; red) and CD8^+^ (bottom; red) T cells expressing Ki67 (green) before and after vaccination. Cumulative data showing the frequency of proliferating CD4^+^ (*B*) and CD8^+^ (*C*) T cells, before (black) and after (gray) vaccination. *D* and *E*, Representative images of CD4 (green) and Foxp3 (red; *D*) and cumulative data showing the frequency of CD4^+^ T cells that were Foxp3^+^ before (black) and after (gray; *E*) vaccination. Data represent a mixture of paired and unpaired samples and were assessed by the Mann-Whitney test. *P < .05.

Subsequently, we investigated whether the increased T-cell accumulation in VZV antigen–challenged skin after Zostavax vaccination was due in part to local proliferation at the challenged site. To do this, we compared the expression of Ki67 within CD4^+^ and CD8^+^ T cells in skin biopsy specimens collected at 3 and 7 days from the site of VZV antigen challenge. There was a trend toward an increase in proliferation in the CD4^+^ T-cell population that was not significant (Figure 4A and 4B). However, there was a significant increase in CD8^+^ T-cell proliferation at both 3 and 7 days after VZV antigen challenge in the skin of individuals who had received the Zostavax vaccine (Figure 4A and 4C). This suggests that, in addition to recruitment from the blood, Zostavax vaccination increases the proliferation of T cells, especially the CD8^+^ subset, at the site of VZV antigen challenge, which contributes to increased T-cell accumulation.

Although the frequency of Foxp3^+^ Tregs was unaltered in unchallenged skin after Zostavax vaccination (Figure 2C and 2D), the proportion of Foxp3^+^ Tregs significantly decreased at the site of VZV antigen challenge after vaccination when compared to prevaccination VZV antigen challenge responses (Figure 4D and 4E). This was not due to an absolute decrease in Treg numbers but instead was due to an increase in the non-Treg CD4^+^ T-cell population. This suggests that Tregs either proliferate to a lesser extent in the skin than conventional T cells or are not recruited to the same extent from the blood after vaccination.

### Transcriptional Analysis After Zostavax Vaccination

**Figure 5.**
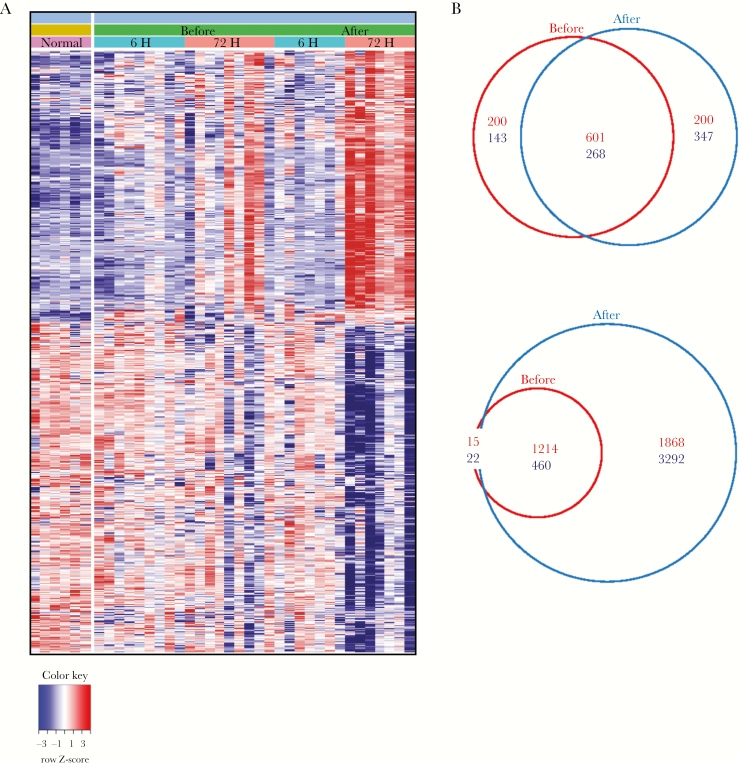
Transcriptomic analysis of varicella zoster virus (VZV) antigen-challenged skin before and after Zostavax vaccination. Punch biopsy specimens (diameter, 3 mm) were collected from older individuals before and after vaccination at 6 and 72 hours after VZV antigen challenge (n = 9 before; n = 7 after). Unchallenged skin punch biopsy specimens were collected from an additional group of volunteers (n = 6). Total skin RNA was isolated, amplified, and hybridized to Affymetrix Human Genome U133 2.0 plus arrays. *A*, Heatmap showing the relative expression of differentially expressed genes (DEGs) between VZV antigen–challenged and normal unchallenged skin before (left panel) and after (right panel) vaccination at a fold change of >2 and a false-discovery rate of >0.05. (B) Venn diagrams showing numbers of DEGs at 6 hours (top) and 72 hours (bottom) following VZV antigen challenge, compared with normal unchallenged skin. Upregulated genes are shown in red, and downregulated genes are shown in blue.

We next performed global gene expression analyses to identify genes that may be associated with the improved response to VZV antigen challenge following vaccination. For each donor, skin punch biopsy specimens were collected from the site of VZV antigen challenge (at 6 hours and 72 hours after challenge), both before and after Zostavax vaccination. The gene expression was compared to that in biopsy specimens collected from unchallenged skin. In a previous study, we showed that, in unchallenged skin, there was little difference in gene expression between young and older age groups [8]. Six hours after VZV antigen challenge, there was a relatively minor difference in the numbers of differentially expressed genes (DEGs) before and after Zostavax vaccination (Figure 5A and 5B and Supplementary Table 1). Genes associated with migration (eg, *SELL, CXCL10, SELE, ICAM1,* and *IL8*) were among the most upregulated genes in both prevaccination and postvaccination groups as compared to findings in unchallenged skin biopsy specimens, although expression was relatively higher after vaccination (Supplementary Tables 1 and 2).

**Figure 6.**
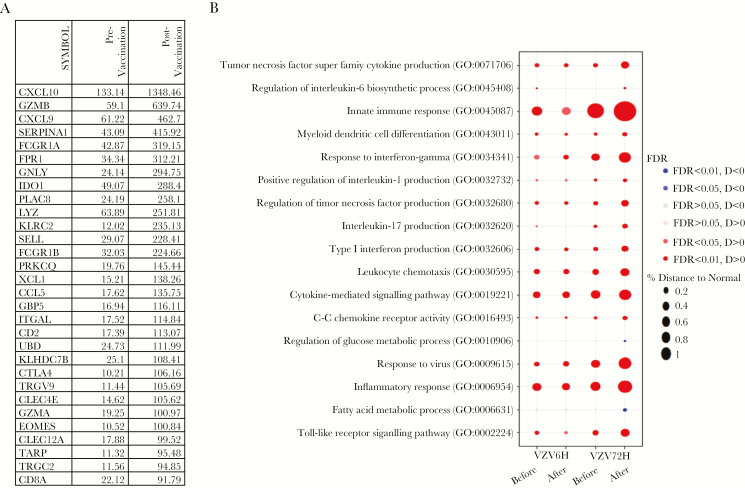
Pathways upregulated in the skin after Zostavax vaccination. *A*, Table showing the top 30 genes upregulated at 72 hours after varicella zoster virus (VZV) challenge before and after vaccination. *B*, Kyoto Encyclopedia of Genes and Genomes and Gene Ontology collection, as well as a curated skin-related collection, were interrogated, and the most relevant pathways among them with a false-discovery rate (FDR) of <0.05 are presented. Bubble plot representing the pathways upregulated in the skin at 6 hours and 72 hours after VZV challenge, before and after Zostavax vaccination. The area of each circle is proportional to the differences in the gene set variation analysis–derived pathway scores between VZV-challenged and unchallenged skin in each group. Colors indicate the direction of dysregulation (red, up; blue, down). Color intensity represents the strength of the dysregulation determined by the FDR.

At 72 hours after VZV antigen challenge, Zostavax vaccination led to a strong transcriptional response that was considerably more pronounced than the 72-hour transcriptional response before vaccination (>5000 DEGs after vaccination [false-discovery rate of <0.05; fold change of >2], compared with 1711 DEGs before vaccination Figure 5A and 5B). The top 30 significantly DEG following Zostavax vaccination are shown in Figure 6A, indicating that the same genes were upregulated 72 hours after VZV antigen challenge but to a significantly higher extent following vaccination. Genes associated with migration (*CXCL10, CXCL9, SELL, CCL5,* and *ITGAL*) and T-cell and dendritic cell activation, including *ITGAX* (which encodes CD11c), *CD2, CD28*, *CD69, EOMES, ICOS*, *STAT1*, and *GZMB,* were more highly expressed in the skin 72 hours after VZV antigen challenge in vaccinated individuals as compared to unvaccinated individuals (Figure 6A). This supports the histological observations of an increase in T-cell accumulation in VZV-challenged skin after vaccination (Figure 3). These data also showed marked upregulation of genes involved in signaling pathways associated with innate immune responses, inflammation, antiviral immune responses, and inflammatory cytokine signaling (type I IFN, TNF-α, and IFN-γ) in vaccinated older individuals (Figure 6B). Of interest, many genes associated with the activity of CD8^+^ cytotoxic T cells, including *GZMB, GNLY, GZMA*, and *CD8A*, were highly induced in vaccinated individuals, coinciding with the significantly increased CD8^+^ T-cell proliferation at the site of VZV antigen challenge at 72 hours (Figure 4C). This indicates that Zostavax vaccination enhances the activity of VZV-specific CD8^+^ T cells in the skin.

## DISCUSSION

The impact of Zostavax vaccination on immunity has typically been assessed by the measurement of anti-VZV antibody levels or changes in VZV-specific T cells in the circulation [13, 27, 30]. Although the morbidity associated with VZV reactivation is associated with the skin, there is little information on the effect of this vaccine on cutaneous immunity. We confirmed findings from previous studies showing that vaccination of older volunteers led to a significant improvement in the cutaneous immune response to VZV [22, 31, 32]. However, using histological and transcriptomic analyses, we extended these observations by characterizing changes in immune cell populations at the cutaneous site of VZV antigen challenge. The first conclusion from our study is that Zostavax vaccination did not, on its own, have a direct impact on the frequency of VZV-specific CD4^+^ T_RM_ in the skin. However, as reported previously, increases in the proportion of VZV-specific CD4^+^ T cells in the circulation were observed [27]. Therefore, Zostavax vaccination protects against HZ by a mechanism that does not involve a direct increase in the frequency of VZV-specific CD4^+^ T_RM_ in the skin. It is possible, however, that the function of VZV-specific T_RM_ may be enhanced by vaccination, either by direct immune boosting or by a dynamic equilibrium between recently activated VZV-specific T cells in the blood and the VZV-specific T_RM_ populations.

Zostavax boosted the clinical response to VZV antigen challenge in older individuals, and this correlated with increased endothelial activation and enhanced CD4^+^ T-cell accumulation in VZV antigen–challenged skin. These findings support our data from young individuals, in whom VZV antigen challenge resulted in early endothelial activation beginning 6 hours after challenge and peaking by 24 hours, while T-cell accumulation was observed by day 3 and peaked around day 7 [12, 18]. Collectively, these data suggest that, in vaccinated older individuals, the improvement in clinical response to VZV antigen challenge is dependent on an enhancement in early endothelial activation, which leads to increased T-cell recruitment to the challenged site.

Previous studies have reported an increase in Foxp3^+^ Tregs in normal unchallenged skin during aging [12, 19, 33]. Furthermore, in aging individuals there is a highly significant correlation between an increased proportion of Tregs in the skin and decreased immune responses to VZV antigen challenge [12]. It is likely that the increased proportion of Tregs in skin may hinder the activation of T_RM_ during VZV antigen challenge in older individuals. During the time course of a cutaneous immune response to VZV antigen challenge in young individuals, the numbers of CD4^+^ Tregs increased in parallel with conventional CD4^+^ T cells so that the proportions of these cells within the CD4^+^ compartment remained relatively stable [19]. In contrast, after Zostavax vaccination the proportion of CD4^+^ Tregs in the skin decreased significantly, which may explain why the cutaneous immune response to VZV antigen challenge increased in vaccinated individuals. However, the total number of Tregs was relatively unchanged in VZV-challenged skin after Zostavax vaccination, suggesting that either the rate of proliferation of Tregs in the skin is lower than that of conventional T cells or that conventional T cells are preferentially recruited from the circulation. It is unlikely that Foxp3 in this case represents a marker of activated cells, as our previous work has shown that these Foxp3^+^ cells are suppressive [19]; furthermore, there is no significant overlap in the expression of Foxp3 and the proliferation marker Ki67. One hypothesis is that, since Zostavax vaccination increases the proportion of VZV-specific CD4^+^ T cells in the blood, the recruitment of these cells into the skin during VZV antigen challenge amplifies the immune response, induces optimal endothelial activation, and overrides the inhibitory influence of the cutaneous Treg populations in older individuals.

Another conclusion is that Zostavax vaccination induces a strong CD8^+^ cytotoxic T-cell–related gene signature in the skin after VZV antigen challenge, which occurs in parallel with CD8^+^ T-cell proliferation in situ. In a previous study, while circulating CD4^+^ T-cell responses were observed in response to multiple VZV antigens, only ORF-9 induced significant CD8^+^ T-cell responses. Zostavax vaccination led to 2.8-fold and 4.9-fold increases in the frequency of ORF-9–specific CD8^+^ T cells producing IFN-γ and TNF-α, respectively [15]. The enhanced CD8^+^ T-cell signature in the skin after VZV antigen challenge in vaccinated older individuals indicates that it will be important to determine the specificity of these cells and their role in the protection against HZ.

Finally, a new VZV glycoprotein E subunit vaccine (Shingrix) has been developed that provides >97% protection against the development of HZ in older individuals [34], which compares highly favorably to Zostavax, which was only 37.6% effective in individuals aged >70 years [13]. It will be of considerable interest to determine the impact of Shingrix on cutaneous immunity after VZV antigen challenge, especially on the CD8^+^ T-cell compartment, and to compare the transcriptional response in VZV-challenged skin after Shingrix vaccination to that induced by Zostavax. This may identify correlates of immune enhancement and pathways that may be manipulated to boost immunity during aging.

## Supplementary Data

Supplementary materials are available at *The Journal of Infectious Diseases* online. Consisting of data provided by the authors to benefit the reader, the posted materials are not copyedited and are the sole responsibility of the authors, so questions or comments should be addressed to the corresponding author.

Supplementary Table 1

Supplementary Table 2

## Notes

***Acknowledgments.*** We thank Dr Azharul Mannan and Dr Simon Oakley, for discussions; all of the volunteers who participated in this study; And our research nurses, Ms Jo Williams and Ms Megan Harries, for their support in volunteer recruitment.

***Financial support.*** This work was supported by the British Skin Foundation, Dermatrust, the Medical Research Council (Grand Challenge in Experimental Medicine Grant MR/M003833/1 to A. A., M. V. S., and N. A. M.), and the Biotechnology and Biological Sciences Research Council (Institute Strategic Programme Grant BB/BB/P013740/1 to N. A. M.).

***Potential conflicts of interest.*** All authors: No reported conflicts of interest. All authors have submitted the ICMJE Form for Disclosure of Potential Conflicts of Interest. Conflicts that the editors consider relevant to the content of the manuscript have been disclosed.

***Supplement sponsorship.*** This work is part of a supplement sponsored by xxx.
