# Supplementary material for: Impact of Zostavax Vaccination on T-Cell Accumulation and Cutaneous Gene Expression in the Skin of Older Humans After Varicella Zoster Virus Antigen–Specific Challenge
Source: J Infect Dis. 2018 Sep 22;218(Suppl 2):S88–98. doi: 10.1093/infdis/jiy420 (PMC6151076; doi:10.1093/infdis/jiy420)
Supplement: Supplementary Table 2 [file jiy420_suppl_supplementary_table_2.docx]

|  | PRE VAC | POST VAC |
| --- | --- | --- |
| GENE | FCH VZV vs Normal skin | |
| CXCL10 | 49.8 | 525.5 |
| IL6 | 42.67 | 58.91 |
| IL1B | 38.03 | 49.51 |
| FPR1 | 63.83 | 48.5 |
| SELE | 28.84 | 48.07 |
| IL8 | 18.36 | 46.99 |
| CXCL2 | 18.88 | 46.92 |
| PTX3 | 40.04 | 45.88 |
| SERPINA1 | 39.72 | 44.44 |
| FOSL1 | 27.09 | 43.13 |
| FCGR1A | 56.43 | 42.88 |
| CXCL1 | 22.1 | 39.46 |
| OASL | 27.39 | 38.96 |
| FCGR1B | 49.94 | 35.94 |
| BCL2A1 | 26.49 | 33.14 |
| IRF1 | 13.23 | 29.32 |
| ICAM1 | 17.93 | 29.07 |
| RGS16 | 12.93 | 27.56 |
| *MMP12* | *11.13* | *24.01* |
| SELL | 15.58 | 22.32 |
| UBD | 13.2 | 22 |
| CXCL9 | *6.01* | 21.91 |
| CH25H | 11.71 | 21.4 |
| CCL3 | 13.39 | 21.28 |
| CCL8 | 14.33 | 19.65 |
| CCL2 | 13.48 | 18.78 |
| IL1RL1 | 16.94 | 18.37 |
| RGS16 | 8.89 | 17.8 |
| GZMB | 11.41 | 17.55 |
| ADAMTS8 | 12.22 | 16.88 |

**Supplementary Table 2: A List of the top 30 genes upregulated at 6 hours post VZV challenge**
